# The Use of Lithium in Veterinary Practice*Read before the Illinois State Veterinary Medical Association.

**Published:** 1892-04

**Authors:** S. S. Baker


### THE USE OF LITHIUM IN VETERINARY PRACTICE. *

By S. S. Baker, D. V. S.

In casting about for a subject for your consideration, that was not already worn threadbare, it occurred to me that a remedy I had been using of late, with a good deal of success, might be of interest to you. The use of lithium has only of late been introduced to any extent in medical practice, and not at all in veterinary practice, as far as I have been able to discover, and why it has remained an obscure and untried drug so long I am at loss to understand. I certainly have found it a very valuable addition to our list of remedies, its only objection being its expense.

Lithium, like the potassium salts, are of an alkaline nature, and while the two salts are alike, to a certain degree, they differ very materially in some of their actions ; the principal difference being the solvent power of lithia over uric acid, with which it forms a very solvent salt, while the potassium salts have no such power. There are five salts of lithium that are officinal, viz., the

* Read before the Illinois State Veterinary Medical Association.

bromide, benzoate, salicylate, carbonate and citrate ; the two last are the only ones applicable to our patients.

While the carbonate has an alkaline reaction, it is nearly insoluble, taking 130 parts of water at 59° F., and about the same amount of boiling water, is wholly insoluble in alcohol. Therefore, this salt is only available when given in powder or bolus. The citrate of lithium is very soluble, dissolving in 5.5 water at 59° F., therefore, this salt is the one I wish more particularly to call your attention to. While the citrate has a neutral reaction, and is useless as a local alkali for the neutralization of acids, it is transformed to a carbonate, after becoming absorbed into the blood; constitutionally, therefore, it is the equivalent of the carbonate, and is finally eliminated by the kidneys. While the salts of lithium are, as the potassium salts, powerful diuretics, they are less irritant to the stomach and kidneys, and on this account are far preferable in the treatment of the disease I am using it for, than the potassium salts. The disease that this drug is the most applicable, is in the treatment of Azoturia, and to my mind it far excels any other drug for that purpose. I will cite a few cases where I have tried it to my utmost satisfaction :

Case I. Seven year old truck horse, taken with azoturia four miles from home ; the driver noticing something wrong with him, got him unhitched and into a livery stable before he went down. I got to him one hour afterwards, found him unable to rise, sweating profusely, and struggling furiously. After passing catheter, I prescribed lithii citras in doses of 2 drachms, together with tinct. gentian, every three hours. Next day found the horse able to pass urine, and making very good efforts to rise. I reduced the lithium to 1 drachm doses three times a day. Next day I found the horse standing, but moved about with difficulty. I continued the lithium for two days longer, when I changed to nux vomica, etc. The horse walked home in ten days.

Case II. Driving mare nine years old brought to hospital, having been taken close by ; she went down immediately after being admitted, passed catheter, and gave lithii citras in 2 drachms doses every three hours for twenty-four hours. Second day mare got up and passed urine without the aid of catheter; reduced the doses of lithium to 1 drachm three times a day for two days longer. When lithium was stopped, and cinchona, etc., was given, the mare made a good recovery in a week.

Case III. I consider this case of the most importance, as demonstrating the applicability of this drug in the treatment of this disease. I was called to see a six-year-old cab horse, suffering with what was supposed to be colic. I received the call at 9 o'clock P. M., and found that the horse had been down since 5 o'clock that afternoon, the owner thinking it a case of colic, from which he would recover without assistance. I found the horse in a profuse perspiration, unable to rise, although making frantic efforts to do so. I found considerable hyperæmia of the muscles of the lumbar region; some fever; pulse rapid and hard, with considerable delirium after passing catheter. I prescribed lithii citras in doses of 2 drachms every three hours, and bromides very three hours alternately; applied hot salt to the loins. Next morning I found the delirium gone, and horse resting easily; the attendant thought the horse had urinated, but passed catheter; and found that he had not stopped the bromides, still continuing the lithia, with the addition of tinct. gentian; passed the catheter again at night. Next day the attendant was positive the horse had urinated, consequently did not pass the catheter again. The horse remained down ten days before being able to rise; when he would stand a few minutes and lie down again. I continued the lithium every three hours for four days, when I changed it to three times a day in 1 drachm doses, with the addition of cinchona and nux. One week from the time he got up he walked about, but was very lame. He improved rapidly, and went to work in about six weeks from time he was first taken.

Since beginning the use of lithium I have had a number of cases of this malady, with only one fatal result; a draft mare being brought to the hospital in an ambulance; she was dragged to box stall on her side and expired four hours afterwards. While not claiming that this drug is a specific in this trouble, it has certainly given me more satisfaction than any other I ever used.

If in bringing this subject to your notice it is the means of assisting any brother practitioner in the treatment of this, the bugbear of all diseases to the profession, I will feel that my efforts have not been in vain, and I would be most pleased to hear with what success any of you may meet, after trying this drug.

